# Hypoplastic Internal Carotid Artery Co-Presenting with Neurofibromatosis and Intracranial Masses

**DOI:** 10.7759/cureus.750

**Published:** 2016-08-26

**Authors:** Arvin R Wali, David R Santiago-Dieppa, Jeffrey A Steinberg, Ali Alattar, Vincent J Cheung, Royya Modir, Alexander A Khalessi, J. Scott Pannell

**Affiliations:** 1 Department of Neurosurgery, University of California, San Diego; 2 School of Medicine, University of California, San Diego

**Keywords:** neurofibromatosis, optic glioma, hypoplastic internal carotid artery, lentiform mass, nerve sheath tumor, neurovascular abnormalities, intracranial vascular malformations, aplastic internal carotid artery, intracranial mass, neurofibromin

## Abstract

Neurofibromatosis type 1 (NF1) is associated with systemic vascular disease, and it can also affect intracranial vasculature in a small percentage of patients. Very rarely, NF1 may co-present with hypoplasia of the internal carotid artery (ICA). Prior reports have documented NF1 with bilateral optic gliomas and a unilateral hypoplastic internal carotid artery; however, we report a case with the aforementioned findings in addition to a right-sided lentiform mass. This case report further suggests a common congenital pathway related to neurofibromin loss of function resulting in both nerve sheath tumors and cerebrovascular anomalies.

## Introduction

Neurofibromin loss of function can result in systemic malignancies and vascular abnormalities [1]. Neurofibromatosis type 1 (NF1) affects one in three thousand people and can be associated with systemic vascular disease and malformations affecting the aortic, renal, and mesenteric arteries [2-3]. On the other hand, hypoplasia or aplasia of the internal carotid artery (ICA) not secondary to mass effect or compression is another very rare congenital anomaly that may lead to neurologic deficits or life threatening intracranial vascular abnormalities such as aneurysm formation [4-6]. 

Case series have demonstrated NF1 with non-specific cerebrovascular dysplasia in as few as two percent of patients [7-9]. Moreover, in a case series reviewing NF1 and a hypoplastic ICA, the presence of optic gliomas was found in only a third of patients [10]. Here we discuss an extremely rare case that demonstrates a hypoplastic ICA, NF1, bilateral optic gliomas, and an additional lentiform mass. Informed consent was obtained from the patient for this study.

## Case presentation

**Figure 1:**
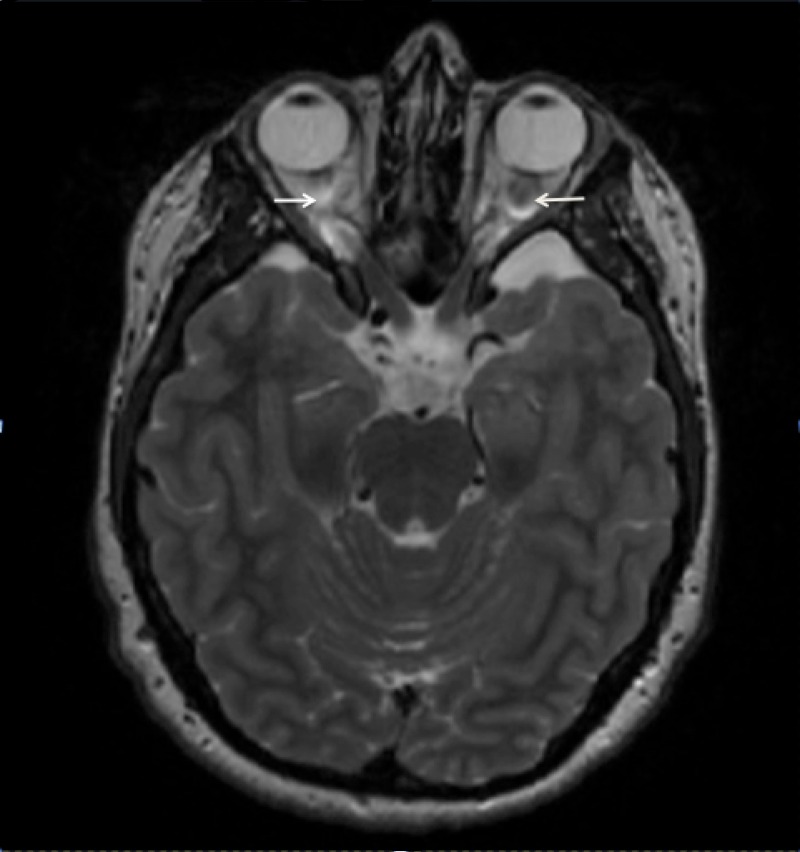
T2 weighted axial MRI through the suprasellar cistern Bilateral optic nerve enlargement consistent with optic nerve gliomas. No significant mass effect on adjacent vasculature.

**Figure 2:**
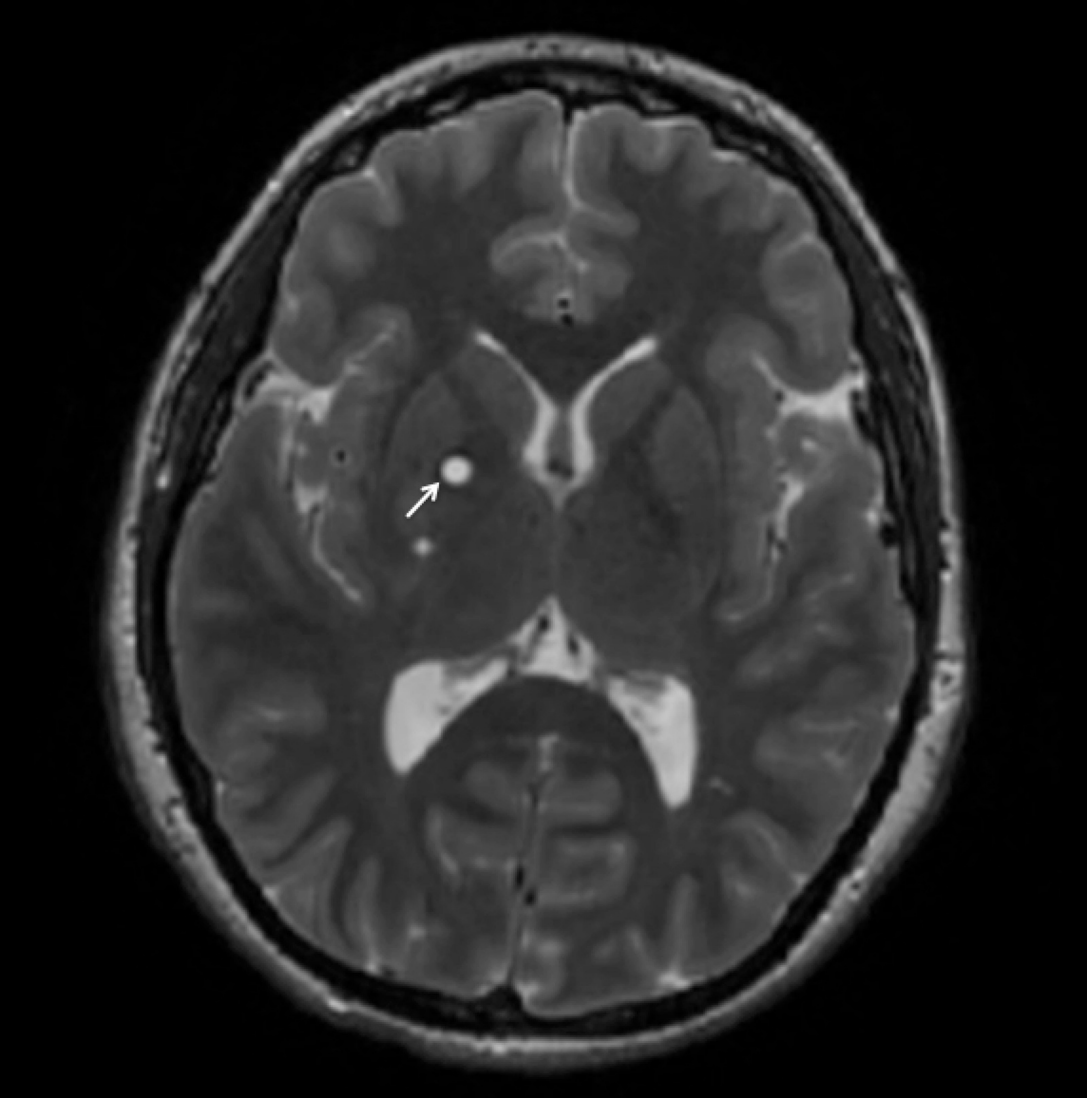
Axial T2 FLAIR MRI Hyperintense lesions within the right lentiform nucleus concerning for glioma.

**Figure 3:**
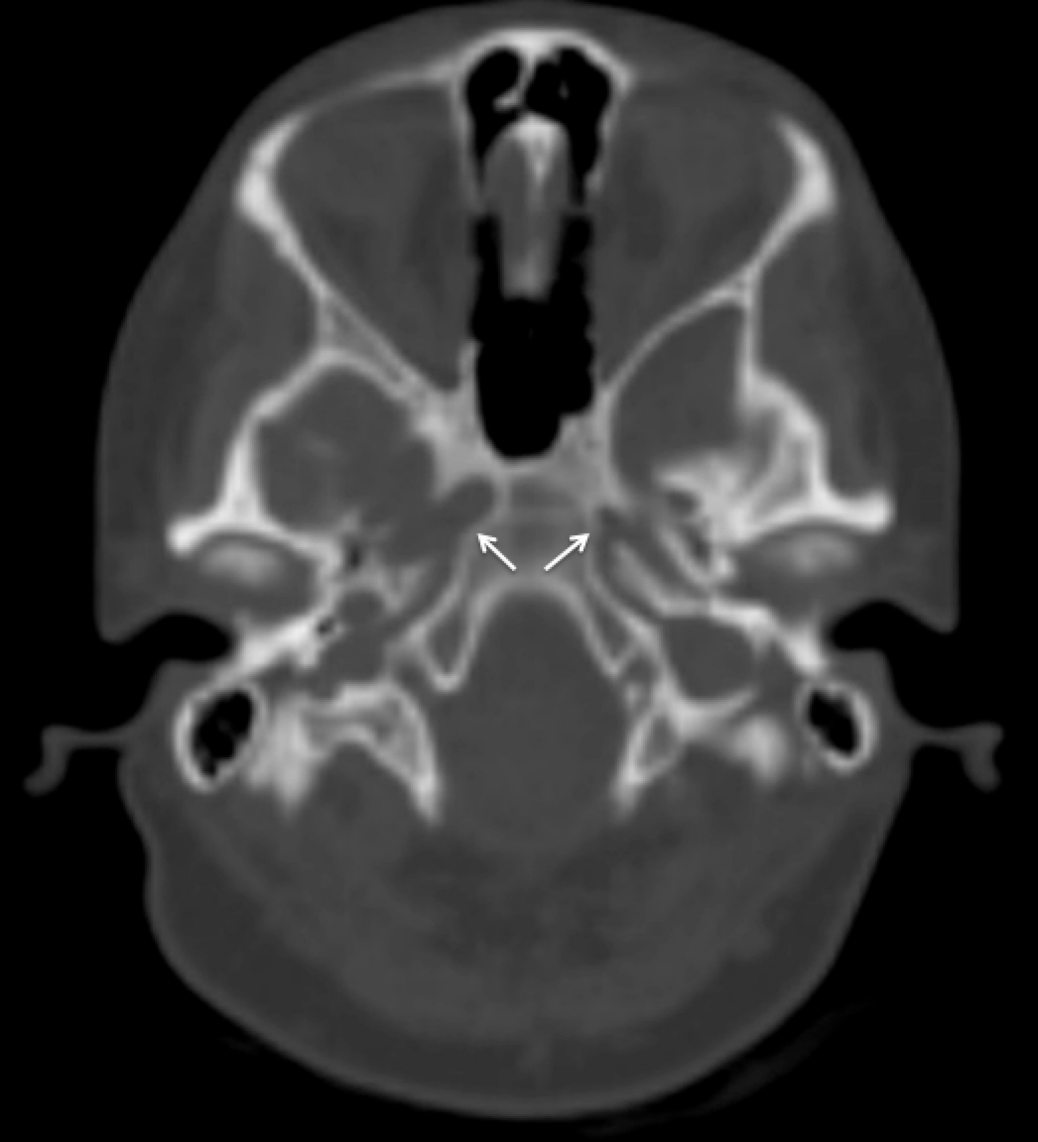
Axial CT The right carotid canal and left hypoplastic carotid canal are demarcated by arrows.

Our patient is an 18-year-old female with known NF1 and bilateral optic gliomas who presented to the neurology department for evaluation of a hypoplastic left ICA demonstrated on a prior CT angiogram at an outside hospital. Given the rarity of a hypoplastic ICA in NF1 patients, workup was initiated with particular attention given to alternate etiologies such as Moyamoya syndrome. The patient had a long-standing history of headaches, chronic left-greater-than-right vision loss, intermittent vertigo, numbness, tingling, and pain. A physical exam confirmed her visual symptoms and demonstrated 4+/5 right upper extremity weakness, hyporeflexia of the left triceps reflex, and diffuse café au lait spots. The patient underwent magnetic resonance imaging (MRI) and magnetic resonance angiography (MRA) imaging of the brain (Figures 1-3).

**Figure 4:**
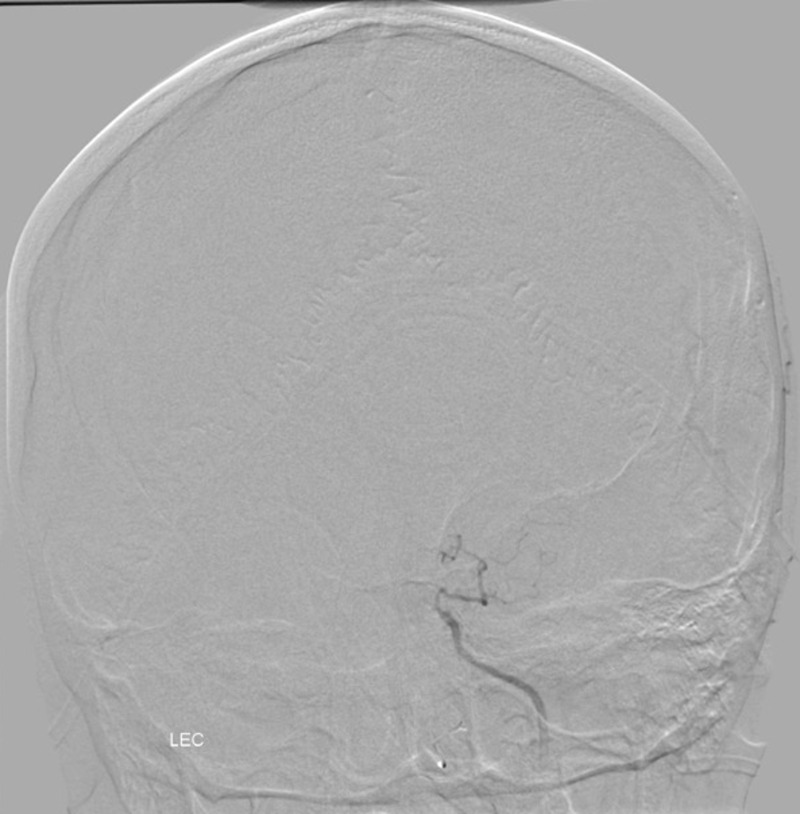
Posterior anterior (PA) angiogram of the anterior circulation Left ICA demonstrates absence of the intracranial left ICA distal to ophthalmic branch consistent with hypoplasia of the left ICA.

**Figure 5:**
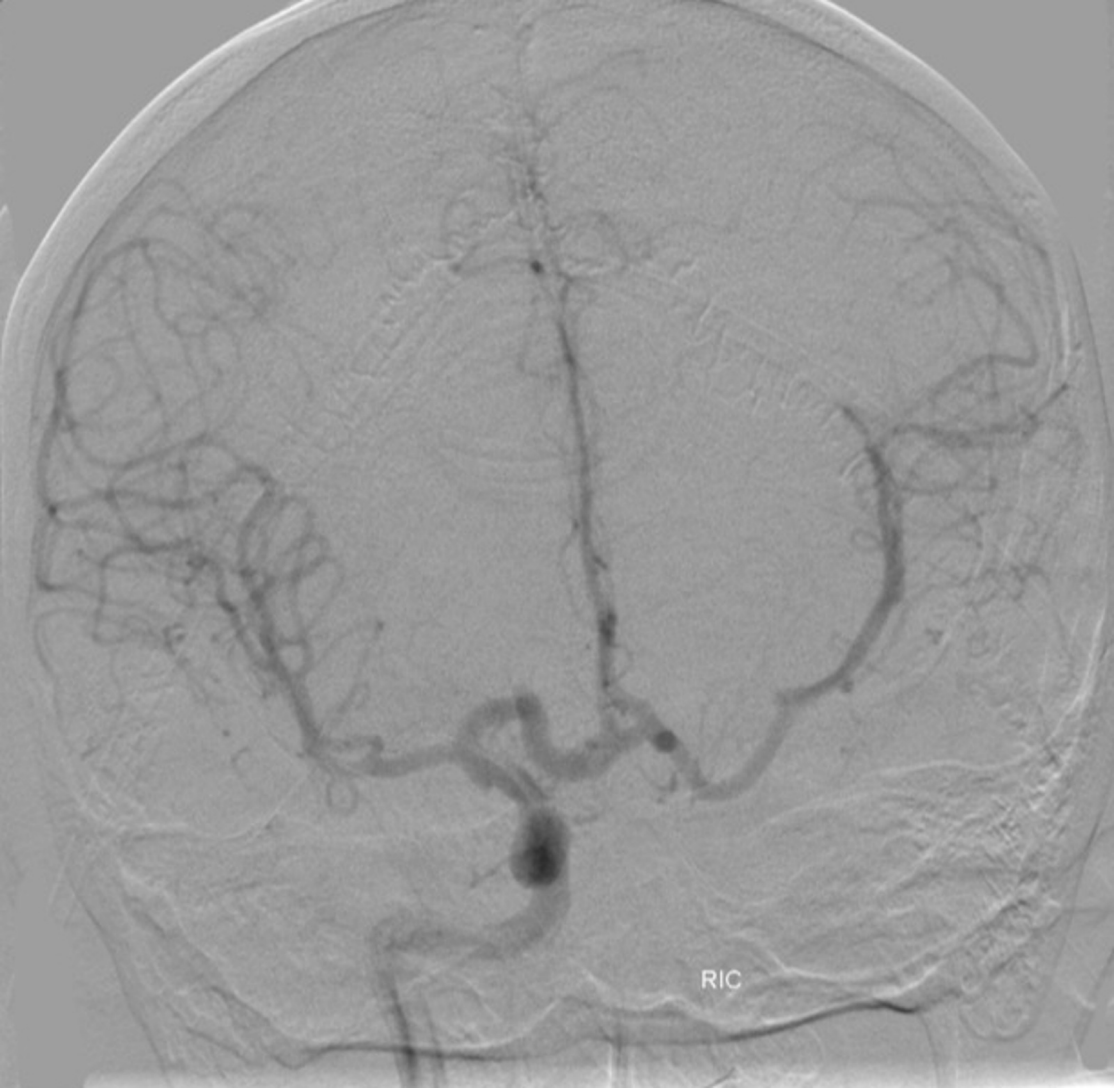
PA angiogram of the anterior circulation Opacification of the bilateral middle cerebral and anterior cerebral arteries via enlarged anterior communicating artery.

The patient then underwent a diagnostic six-vessel cerebral angiogram (DCA) that demonstrated a complete left internal carotid occlusion distal to the branch of the ophthalmic artery. In addition, the entire left middle cerebral artery (MCA) and anterior cerebral artery (ACA) vascular territories were supplied by the right internal carotid via a robust anterior communicating artery (Figures 4-5).

The findings on the DCA excluded the diagnosis of Moyamoya syndrome and further characterized the complete occlusive disease of her left carotid artery with total perfusion of her anterior circulation via the right ICA through a very robust anterior communicating artery. Moreover, a computerized tomography (CT) of her head revealed hypoplasia of the internal carotid canal suggesting a long-standing congenital lesion. The additional lentiform mass was radiographically determined as not exerting mass effect and warranted scheduled surveillance imaging without further intervention. The patient has since been conservatively followed by the neurology service.

## Discussion

This case demonstrates the presence of an intracranial mass in addition to a hypoplastic ICA and optic gliomas in an NF1 patient. This constellation of findings is extremely rare and further demonstrates a clinical example in which loss of function in neurofibromin results in both intracranial and systemic nerve sheath tumors and congenital vascular anomalies. This clinical presentation highlights the importance of further understanding the genetic pathways leading to neurologic tumors and vascular abnormalities. Moreover, this case suggests that further screening for intracranial or congenital malformations in the setting of neurofibromatosis is warranted to optimize management of vascular risk factors to minimize vascular neurologic injury.

## Conclusions

Although neurofibromatosis is exceedingly rare, this disease can co-present with vascular manifestations, and this case report suggests the workup of congenital and acquired intracranial vascular disease for this patient population.
